# Exploring the social context and origins of lifetime trauma in individuals with dementia, and its implications for dementia care

**DOI:** 10.1002/alz70858_101836

**Published:** 2025-12-25

**Authors:** Liam Franco Borelli‐Millott, Sarah Bendall, Monica Cations, Dennis Velakoulis, Anita M Y Goh

**Affiliations:** ^1^ The University of Melbourne, Parkville, VIC, Australia; ^2^ National Ageing Research Institute, Parkville, VIC, Australia; ^3^ Orygen, Parkville, VIC, Australia; ^4^ Flinders University, Adelaide, SA, Australia; ^5^ Neuropsychiatry Centre, The Royal Melbourne Hospital, Parkville, VIC, Australia; ^6^ University of Melbourne, Parkville, VIC, Australia

## Abstract

**Background:**

An individual's life history and past trauma may have implications for their experience of dementia and dementia care relationships. However, an understanding of the social context and origins of trauma and how these may be present in dementia care is missing from the literature and could be useful when implementing trauma‐informed dementia care practices. The objective of this study was to explore the phenomenology of trauma documented in the psychosocial histories of individuals diagnosed with dementia, and to discuss how the social aspects of that trauma may impact dementia care.

**Method:**

We screened and sampled 113 individuals with younger onset dementia diagnoses that were admitted to a specialist neuropsychiatric health service between 2017 and 2021 (inclusive), using clinical discharge summaries as a data source. Clinician's descriptions of psychiatric and life history were used to identify individuals with a history of trauma. Text descriptions of trauma were extracted and gathered using a data collection tool. The study used qualitative methods, with the extracted text subject to reflexive thematic analysis. This method was used to construct themes encompassing trauma experienced by individuals within the sample. To compliment the qualitative analysis, demographic and clinical data was collected to characterise the sample.

**Result:**

Forty‐four individuals had a documented history of trauma. Ten themes were constructed using reflexive thematic analysis of the data set. The themes encompassed the social origins and context of the past trauma experienced by the sampled individuals. The themes related to dynamics that play out at a personal, interpersonal, familial, historical and societal level.

**Conclusions:**

This study provides further understanding of the experiences of trauma in people living with dementia, and how they are embedded within an individual's social life history and world. The findings demonstrate the potential for trauma to influence current and future dementia care relationships and provide context for how these relationships may currently be playing out within healthcare systems that provide dementia care. The findings can directly inform what should be considered when researching, planning and implementing trauma‐informed care for individuals living with dementia.

